# Acupuncture indication knowledge bases: meridian entity recognition and classification based on ACUBERT

**DOI:** 10.1093/database/baae083

**Published:** 2024-08-30

**Authors:** TianCheng Xu, Jing Wen, Lei Wang, YueYing Huang, ZiJing Zhu, Qian Zhu, Yi Fang, ChengBiao Yang, YouBing Xia

**Affiliations:** Key Laboratory of Acupuncture and Medicine Research of Ministry of Education, Nanjing University of Chinese Medicine, 138 Xianlin Road, Nanjing 210023, China; School of Medical Information and Engineering, Xuzhou Medical University, 209 Tongshan Road, Xuzhou 221004, China; Key Laboratory of Acupuncture and Medicine Research of Ministry of Education, Nanjing University of Chinese Medicine, 138 Xianlin Road, Nanjing 210023, China; School of Medical Information and Engineering, Xuzhou Medical University, 209 Tongshan Road, Xuzhou 221004, China; Nanjing KG Data Technology Co., Ltd., 1 Dongji Road, Nanjing 211100, China; Key Laboratory of Acupuncture and Medicine Research of Ministry of Education, Nanjing University of Chinese Medicine, 138 Xianlin Road, Nanjing 210023, China; School of Medical Information and Engineering, Xuzhou Medical University, 209 Tongshan Road, Xuzhou 221004, China; Key Laboratory of Acupuncture and Medicine Research of Ministry of Education, Nanjing University of Chinese Medicine, 138 Xianlin Road, Nanjing 210023, China; School of Medical Information and Engineering, Xuzhou Medical University, 209 Tongshan Road, Xuzhou 221004, China; School of Medical Information and Engineering, Xuzhou Medical University, 209 Tongshan Road, Xuzhou 221004, China; Department of Traditional Chinese Medicine, Medical School, Qinghai University, 251 Ningda Road, Xining 810016, China; Key Laboratory of Acupuncture and Medicine Research of Ministry of Education, Nanjing University of Chinese Medicine, 138 Xianlin Road, Nanjing 210023, China; School of Medical Information and Engineering, Xuzhou Medical University, 209 Tongshan Road, Xuzhou 221004, China; Nanjing KG Data Technology Co., Ltd., 1 Dongji Road, Nanjing 211100, China; School of Computer Science and Engineering, Southeast University, 2 Dongnandaxue Road, Nanjing 211102, China; Key Laboratory of Acupuncture and Medicine Research of Ministry of Education, Nanjing University of Chinese Medicine, 138 Xianlin Road, Nanjing 210023, China; School of Medical Information and Engineering, Xuzhou Medical University, 209 Tongshan Road, Xuzhou 221004, China

## Abstract

In acupuncture diagnosis and treatment, non-quantitative clinical descriptions have limited the development of standardized treatment methods. This study explores the effectiveness and the reasons for discrepancies in the entity recognition and classification of meridians in acupuncture indication using the Acupuncture Bidirectional Encoder Representations from Transformers (ACUBERT) model. During the research process, we selected 54 593 different entities from 82 acupuncture medical books as the pretraining corpus for medical literature, conducting classification research on Chinese medical literature using the BERT model. Additionally, we employed the support vector machine and Random Forest models as comparative benchmarks and optimized them through parameter tuning, ultimately leading to the development of the ACUBERT model. The results show that the ACUBERT model outperforms other baseline models in classification effectiveness, achieving the best performance at Epoch = 5. The model’s “precision,” “recall,” and *F*_1_ scores reached above 0.8. Moreover, our study has a unique feature: it trains the meridian differentiation model based on the eight principles of differentiation and zang-fu differentiation as foundational labels. It establishes an acupuncture-indication knowledge base (ACU-IKD) and ACUBERT model with traditional Chinese medicine characteristics. In summary, the ACUBERT model significantly enhances the classification effectiveness of meridian attribution in the acupuncture indication database and also demonstrates the classification advantages of deep learning methods based on BERT in multi-category, large-scale training sets.

**Database URL**: http://acuai.njucm.edu.cn:8081/#/user/login?tenantUrl=default

## Introduction

Acupuncture, with a history spanning over two millennia in China, enjoys recognition from 113 World Health Organization (WHO) member countries [1]. Many developed countries, including the USA, have integrated it into their government healthcare insurance systems [2]. UNESCO listed it as an Intangible Cultural Heritage in 2010 [3]. Despite its numerous disease types with significant treatment benefits and proven clinical efficacy, with top journals like *Nature* featuring its mechanisms [4], acupuncture faces significant challenges in standardization due to the diversity of its thousands of years of history and medical practice. Variations in language and individualized documentation methods across different eras and among various medical practitioners make effectively standardizing acupuncture textual data challenging and hinder the analysis and broader application of acupuncture medical knowledge through intelligent tools like ChatGPT.

The two core features of traditional Chinese medicine (TCM) theory are syndrome differentiation and the concept of holism. In acupuncture, the syndrome differentiation method plays an important role and has a widespread use in clinical practice. It covers complex content such as meridian differentiation, zang-fu differentiation, and the eight principles of differentiation, integrating viewpoints from classical texts of various eras. Acupuncture prescriptions adhere to TCM’s holistic code, viewing the human body as a homeostatic system akin to natural ecosystems The dynamic equilibrium of the core elements of the eight principles of differentiation (Yin, Yang, Exterior, Interior, Cold, Heat, Deficiency, and Excess) maintains this system’s balance. Practitioners often consider diseases or symptoms as results of imbalances among these elements. Therefore, treating specific diseases aims to restore the balance of the body’s homeostatic system, achievable by regulating the meridian pathways with acupuncture points (acupoints). The human body has 14 main meridians, each associated with specific TCM organs (zang-fu), forming a complex physiological regulatory network. These meridians are responsible for transporting qi and blood, which is crucial in maintaining the balance among the eight elements mentioned above. Yin and Yang, the two primary principles, are essential in diagnosing the nature of diseases, reflected in the flow of Qi and blood and the functional states of the zang-fu. The exterior and interior principles reveal the depth of pathological changes and the severity of the condition. Imbalances between cold and heat often originate from Yin and Yang imbalances or external pathogenic factors, directly affecting the flow of Qi and blood in the meridians. The deficiency and excess principles are used to assess the strength of the human body’s vital energy and pathogenic factors. The smooth flow of the meridians directly impacts the balance of these elements within the body, thus affecting the health of the zang-fu. Therefore, in acupuncture treatment, selecting appropriate points to regulate specific meridians helps balance these eight elements and effectively harmonizes the interactions among the zang-fu, thereby restoring health. However, the accuracy of acupoint selection highly depends on precise syndrome identification. As clinical descriptions in acupuncture diagnosis and treatment are usually qualitative rather than quantitative, this limits the standardization of acupuncture methods and the promotion of effective therapies. Accurate syndrome differentiation is crucial for choosing appropriate acupoints. Still, challenges in the acupuncture syndrome differentiation process, such as subjective symptom descriptions and diagnostic variances among different practitioners, make it more difficult to implement standardized treatment protocols. To address these challenges, adopting modern techniques and methods to quantify acupuncture textual data becomes particularly important. In this work, we have combined the efforts of domain experts (annotators with national acupuncture practitioner qualifications) to create the world’s first acupuncture indication knowledge base (ACU-IKD) with TCM characteristics. This enhances the standardization and understanding of acupuncture therapy and lays a solid foundation for future research and applications.

## Related work

Currently, several teams are actively exploring the field of intelligent acupuncture syndrome differentiation. For instance, Zhang *et al*. [5]. employed network analysis techniques to process relational data, but this method only reveals a limited “syndrome-acupoint” relationship in clinical guidance. Wang *et al*. [6]., based on acupuncture textbooks, used complex networks to construct a directed network for syndrome differentiation and acupoint prescription. They found that selecting acupoints for syndromes follows a random macro network-level pattern. In contrast, the pairing of acupoints with syndromes conforms to scale-free network characteristics. Lee *et al*. [7] utilized machine learning techniques to investigate the clinical usage patterns of the five phases in randomized controlled trial data. They discovered that practitioners do not use the five phases equally in treating diseases and that stream and sea acupoints are more likely to exhibit unique properties than other acupoints. Yu *et al*. [8] merged acupuncture theory with network science, innovating a paradigm for acupoint selection research through a critical acupoint mining algorithm rooted in the acupoint network. They based this method on binary synergistic relationships between acupoints. However, it overlooks the complex synergies among multiple acupoints and fails to uncover the deep-seated patterns of each acupoint within the meridian system.

Although these studies have brought innovative methods to the intelligentization of acupuncture syndrome differentiation, they still face methodological limitations. A comparative analysis of representative research by teams domestically and internationally (Table 1) shows that current research on intelligent acupuncture typically focuses on a single disease or data from a limited number of doctors, lacking a comprehensive database covering multiple diseases and languages. This research gap limits the dissemination of acupuncture knowledge and is one of the main driving factors for our study. One major challenge in this research process is the difficulty for machines in accurately understanding the acupuncture indications. This requires technological innovation and a deep understanding and accurate expression of TCM acupuncture. Therefore, building a specialized acupuncture dataset based on TCM theory is crucial for enhancing the standardization of acupuncture and promoting in-depth research and application of acupuncture knowledge by interdisciplinary research teams.

**Table 1.** Comparative study on the intelligence of acupuncture syndrome differentiation

Research institutions	Research project	Research contents	Research result
Kyung Hee University	Statistical inference in acupoint selection: data mining based on random controlled trials [68]	This study employs *z*-core analysis and Bayesian inference to mine data from consultations by 80 doctors on diseases such as depression, dysmenorrhea, and lower back pain. It focuses on investigating the specific corresponding relationships between acupoints and these diseases.	The process of selecting acupoints based on diseases is specific, but the reverse is not true; the acupoint may not be the only treatment point for that disease type.
Kyung Hee University	Characterization of hidden rules linking symptoms and selection of acupoint using an artificial neural network model [69]	The analysis of 232 clinical records from 81 patients using artificial neural networks can achieve relatively accurate acupoint selection based on symptoms, with an accuracy rate of 0.911 and a recall rate of 0.811.	There are 11 hidden nodes between 22 symptoms and 26 acupoints, providing a typical example for the pattern recognition research of acupuncture and moxibustion clinical acupoint selection.
Chengdu University of TCM	Exploration of an algorithm model for clinical syndrome differentiation of acupuncture and moxibustion and moxibustion in treating stroke [70]	The study focuses on meridian differentiation as the main body, with site differentiation as the critical point, guided by the eight principles of differentiation and supplemented by visceral differentiation, to explore the acupuncture “symptom-prescription” algorithm model for stroke.	The study created a 31-bit coding model for stroke differentiation, tested against 50 case studies. Findings revealed high conformity in eight-principle differentiation, moderate site differentiation, and low visceral and meridian differentiation, suggesting the model’s limited reflection of acupuncture’s unique meridian treatment approach.
Liaocheng University	Classification of acupuncture points based on the Bert model [71]	Based on the Bert model, the study constructed a Bert Chinese Acupoint model through retraining and, during the fine-tuning process, incorporated semantic features of acupoints into the acupoint corpus to enhance the semantic characteristics of the acupoints.	The Bert Chinese Acupoint model introduced in this article achieved classification and prediction for 10 acupoints and improved accuracy by 3% over traditional machine learning methods.

The table outlines the progress in the intelligence of acupuncture syndrome differentiation from 2019 to 2024, detailing research institutions, projects, contents, and results in the field.

## Materials and methods

### Constructing the dataset

#### Data collection

The ACU-IKD database, which we developed, encompasses 54 593 entities extracted from 82 foundational texts on acupuncture medicine. These books cover various aspects of acupuncture, providing a wealth of knowledge and practical information on acupuncture (for a detailed list of medical books, please see the Appendix, details can be found in Supplementary Table S1). The construction of this database has been an ongoing effort since 2017, involving the collaborative extraction of data through both manual and machine learning techniques. Preliminary research findings related to these data have already been published in scholarly articles [9–16].

#### Data annotation scheme

In the clinical diagnosis and treatment of TCM acupuncture, the information involved is multidimensional, encompassing elements, such as Yin and Yang, Exterior and Interior, Cold and Heat, Deficiency and Excess, zang-fu, and Meridians. Our study is the first to use fine-grained annotation methods in the data of TCM acupuncture indications. This approach aims to maintain the characteristics of TCM acupuncture while maximizing its accuracy and efficiency, laying the groundwork for uncovering the implicit knowledge in acupuncture treatment. For this purpose, we have used the canonical texts of the TCM and acupuncture community—original texts from the ‘Huangdi Neijing’ (The Yellow Emperor’s Inner Classic) related to the meridians’ pathways, functions, and their relationship with diseases as a foundation. We conducted a detailed breakdown, annotation, and in-depth study of these texts. The specific rules for meridian annotation are as follows.

Pathways and circulation of meridians: We conduct an in-depth study of the paths and circulation of meridians to understand the routes of disease transmission within the human body more accurately. For example, “lower teeth” is a crucial area in the pathway of the Large Intestine meridian, so “pain in the lower teeth” is annotated as a disease of the Large Intestine meridian. Similarly, “upper teeth” are part of the Stomach meridian’s pathway. Hence, “pain in the upper teeth” is annotated as a disease of the Stomach meridian. This method of locating the paths of meridians in anatomy precisely helps understand the mechanisms of disease transmission. It effectively guides the accurate differentiation of causes and locations of diseases in clinical practice.

Relationship between meridians and zang-fu: Meridians are the primary channels connecting the zang-fu organs. They link the organs to each other and connect them to other parts of the body. Each zang-fu organ corresponds to its main meridian and collateral meridian. These meridians and collaterals not only transmit information from the organs but also reflect the health status of these organs. For example, the Lung meridian is directly related to lung-related diseases (such as asthma, cough, and fullness in the chest), and the Spleen meridian is directly related to spleen-related diseases (such as abdominal bloating, epigastric pain, and a feeling of heaviness).

Pathology of Meridian Abnormalities: In TCM theory, meridians are considered channels for circling qi (vital energy) and blood. Abnormalities in the meridians, such as impeded flow of qi and blood, blockages, or functional disorders, can lead to various related symptoms The “Miraculous Pivot· Channel” chapter provides detailed records of the relationships between meridians and specific diseases, including descriptions of terms like “disease caused by disorder of this channel” and “disease of viscera connecting with this channel.” By meticulously dissecting and annotating the symptoms caused by these meridian abnormalities, we can infer potential pathological changes and the related meridians involved based on specific symptoms This method is precious in clinical practice for accurately identifying meridian issues based on symptoms.

Following the rules mentioned above, we successfully categorized all acupuncture indications into “Meridian” attribution. This result encompasses 14 classic and representative meridian categories, rooted in over 2000 years of traditional Chinese medicine acupuncture history and practice, considered “templates” of traditional acupuncture knowledge. Our research, building upon this groundwork, further expanded the scope of annotation by adding five new label categories, namely: zang-fu markers (including eleven labels, five zang labels: Liver, Heart, Spleen, Lung, Kidney; six fu labels: Gallbladder, Small Intestine, Stomach, Large Intestine, Bladder, Sanjiao), eight principles marker dimension 1 (Yin, Yang), eight principles marker dimension 2 (Exterior, Interior), eight principles marker dimension 3 (Cold, Heat), and eight principles marker dimension 4 (Deficiency, Excess). These new labels, all derived from manual annotations by experts in acupuncture, create the conditions for using machine learning technology to analyze and apply acupuncture knowledge deeply.

During the annotation process, we encountered some specific challenges: for entities with multiple attributes, for example, “cough” could belong to both “Yin” and “Yang.” We adopted a strategy of leaving labels blank to avoid misleading classifications. Among all tags, zang-fu is the only category that permits multiple attributes, allowing certain entities to relate to “Lung” and “Heart.” The four major types (Yin–Yang, Exterior–Interior, Cold–Heat, Deficiency–Excess) encompass eight labels. We employed a binary classification method, annotating each entity with only one unique label. Although these four categories of labels have thousands of years of history in clinical and theoretical practice and are relatively distinct, the main issue we faced overall was the sparsity of data. This sparsity presented a significant challenge in building a related database. This fine-grained annotation work enriched the data and laid a solid foundation for a deeper understanding and application of TCM acupuncture knowledge.

#### Data preprocessing

##### Methods and model considerations for short-text classification

The text’s length significantly impacts the model’s training and performance in text classification. Theoretically, the richness of the information content in the text directly affects how much information the model can learn. Models typically extract features from the text, which are then utilized for pattern recognition and decision-making during training [17].

Our research focuses explicitly on textual entities related to acupuncture indications. These texts are often short and have limited information, presenting both a challenge and an innovation opportunity in our work [18, 19]. To tackle this challenge, we compared the dataset we use, the ACU-IKD dataset, with several other publicly available Chinese short-text multi-classification datasets in terms of text length, as shown in Table 2. This comparison enables us to observe the significant differences in length between texts between the ACU-IKD dataset and other datasets. Understanding these length differences is crucial for our selection of the appropriate machine learning model since different models respond differently to the length of the text.

**Table 2.** Comparison of the length of Chinese short-text multi-classification datasets

Datasets	Average length (characters)	Median
ACU-IKD	4	4
THUC News^a^	19	19
VSQ^b^	8.5	9
CHIP-CTC^c^	27	21
KUAKE-QIC^d^	13	11

The average length of a dataset refers to the average number of characters contained in each entity within the dataset. ^a^THUCNews Dataset (THUC News): This dataset was created by filtering historical data from Sina News RSS subscription channels from 2005 to 2011. It contains 14 candidate classification categories and about 740 000 news documents. The titles of these news articles are commonly used in the field of Chinese short-text classification. We selected one title to calculate the average length and median. ^b^Chinese Video Search Engine Video Titles (VSQ): this dataset includes titles from 26 video categories. Available at https://github.com/xubuvd/Short-Text-Classification. ^c^Clinical Trial Screening Criteria Short Text Classification Dataset (CHIP-CTC): The main objective of this evaluation task is to classify clinical trial screening criteria. All text data are obtained from real clinical trials, and the short-text data are sourced from the “Screening Criteria” section in the public information of clinical trials on the Chinese Clinical Trial Registry website. dMedical Search Retrieval Word Intent Classification Dataset (KUAKE-QIC): This dataset can be accessed at https://tianchi.aliyun.com/dataset/95414. It is used for classifying the intent of search terms in a medical context.

Deep learning models like Bidirectional Encoder Representations from Transformers (BERT) [20] demonstrate their strength through powerful context understanding capabilities. They can capture complex relationships between words when dealing with shorter texts, functioning effectively even with limited text length. Similarly, models like FastText [21], TextCNN [22], BiLSTM + Attention [23], logistic regression [24], random forests [25], support vector machines (SVM) [26], and Bayes [27] classifiers also show their capacity for processing short texts. These models can extract rich contextual information and hidden features from limited text, which plays a significant role in understanding and classifying short texts.

For short-text data, appropriate preprocessing [28] and feature engineering [29, 30] are equally crucial. These techniques include text augmentation [31], such as semantic disambiguation [32] and semantic expansion [33], or enriching the text with derived information through ontologies to reduce sparsity [34]. In some natural language processing (NLP) competitions, we have proven that adding entity annotations significantly improves model accuracy in classifying short texts [35, 36].

Considering that the data for this work is shorter than the typical short-text datasets and poses a more significant challenge to model capabilities, we plan to pretrain the more powerful deep learning model BERT for enhanced information extraction. Through integrating deep learning architectures and the execution of fine-grained data preprocessing pertinent to acupuncture indication, this study endeavors to surmount the constraints imposed by text length and enhance the efficacy of the model’s performance in the classification of acupuncture indication entities.

##### Optimization strategies for datasets with limited feature dimensions

In our project, we face a main challenge where the limited feature dimensions of the prediction data are confined to entity names, leading to a critical issue of a mismatch in data features between the prediction and training sets. As we provide multidimensional features in the training set, while the prediction set is based solely on the single dimension of entity names, we cannot fully utilize the multifaceted features in the training set. Therefore, our strategy shifts to relying exclusively on the core feature of entity names for model training.

To address this challenge, we adopted a specific approach. When the feature distributions of the training and test sets are inconsistent, we attempt to construct a validation set that closely resembles the distribution of the test set. This method aims to ensure the stability of both the training and testing processes, thereby improving the accuracy and reliability of the model. Based on the characteristics of the test set in our project, we chose to remove most features from the training set, retaining only the critical dimension of entity names. This approach is common in many data science competitions; for example, in the 2018 Ant Financial Services Group’s Risk Brain-Payment Risk Identification Competition, the runner-up team employed a similar strategy by removing features that had significant distribution differences between the training and test sets. Generally, text classification tasks also often provide only one dimension of text features. Therefore, we can optimize the dataset and improve the model’s overall performance by fully leveraging deep learning technologies.

##### Multi-label data management methods in single-label multi-classification problems

In our project, aiming to address a single-label multi-classification problem, we encountered a unique situation during the experimental process: some data exhibited multi-label characteristics. We adopted an innovative approach to address this by classifying those multi-label data instances into a separate category, which we could not definitively assign to a single label. We named this the 15th category: Unclear Meridian Attributes. As a result, our dataset now presents a typical single-label, multi-classification problem.

In computer science, distinguishing between multi-label and multi-class problems is crucial. Multi-label problems involve single-data entities corresponding to multiple classification labels [37], representing a “one-to-many” relationship. For instance, a movie might be categorized as a romance or a comedy, where the classification goal is to identify all relevant labels for the entity. In contrast, multi-class problems depict a “one-to-one” relationship, where a single-data entity corresponds to one unique classification label [37]. For example, determining a person’s birthplace involves multiple options, but each individual can belong to only one specific province.

In our research on classifying entities of acupuncture indication, although a single acupuncture indication may be associated with multiple meridian attributes, it seemingly aligns more with the characteristics of a multi-label problem. However, in annotation, clinical diagnosis, and treatment, we prefer to match each acupuncture indication entity with its most significant meridian attribute, thus adopting a multi-classification approach. Our method is akin to herb properties in TCM, where the property of an herb is not an absolute attribute but a dominant quality [38, 39]. Therefore, we adopted a similar approach in this project, matching each entity of acupuncture indication with its most significant meridian attribute, which makes the decision process more explicit and focused, thereby reducing confusion and errors in the analysis and diagnosis process, aligning more closely with the practicalities of TCM practice. Based on this, we ultimately decided to categorize those multi-label data that were difficult to assign to a single meridian attribute into an independent category. Generally, increasing the number of labels can add complexity to the classification task and potentially decrease accuracy. Hence, by classifying complex multi-label data in this manner, not only did we simplify the classification task, but we also helped improve overall classification accuracy. This approach effectively addresses the challenges posed by multi-label data, ensuring the efficiency and accuracy of our model.

##### ACUBERT model architecture

In our research, we developed ACUBERT, a model based on the BERT architecture, to classify entities. We designed it to identify and classify acupuncture indication entities and attribute them to corresponding “Meridian” categories. We chose BERT as the foundation because it has demonstrated exceptional capabilities across various natural language processing tasks. Its main strength is understanding and handling complex terminology and concepts in the medical field, and it allows for fine-tuning for different tasks and domains [40, 41]. Additionally, BERT’s deep bidirectional structure solves a series of challenges in our research, including handling short texts [42], addressing data sparsity [43], dealing with limited feature dimensions [44], and performing single-label multi-classification tasks [45]. The powerful representation learning ability of the BERT model indicates its potential to handle these specific issues, offering a solid technical foundation for our research objectives. The ACUBERT model mainly consists of three layers:

Input Representation: The model receives a sequence of acupuncture indication entities, meticulously labeled with fine-grained tags, as input. A unique [CLS] token, representing the aggregate state of the entire sequence, starts each sequence. Each entity in the sequence is combined with its fine-grained label to form a compound word, which is then processed through BERT’s WordPiece embedding to generate an initial sequence of word embedding vectors E1, E2, .., EN.

ACUBERT Layer: These embedding vectors are then passed to the post-training ACUBERT layer. Here, each layer of the BERT model encodes the input word embeddings, generating a new sequence of semantically related hidden states T1, T2, .., TN. These hidden states capture the complex interactions between words and provide a rich semantic representation for each word.

**Figure 1.**
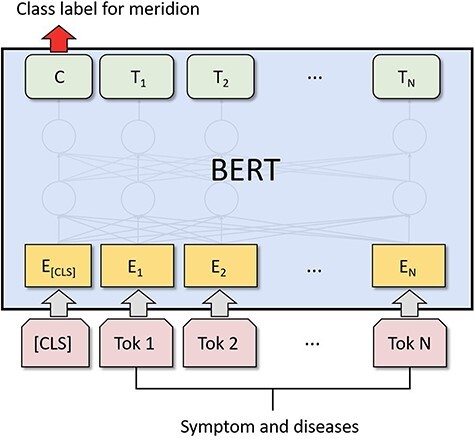
Architecture of the ACUBERT model for meridian classification.

Entity Classification Layer: At the top of the BERT structure, we integrated a layer designed explicitly for entity classification. The purpose of this layer is to map the input entities to 15 different meridian categories. Using the final hidden state C of the first [CLS] token in the sequence, we predict the corresponding meridian category for the entire sequence through a fully connected layer and softmax function. The structure of the ACUBERT model is shown in Fig. 1.

##### Experiments

###### Evaluation indicators

In classifying the “Meridian” attribution of acupuncture indication entities, the goal is to predict the meridian category to which a given acupuncture indication entity belongs. In this experiment, we evaluated the Accuracy (Acc), Precision (P), Recall (R), and F1 score of the classification on the test data using a confusion matrix. True Positives (TP) refer to the number of correctly identified positive acupuncture indication samples for a given meridian; False Positives (FP) are the number of positive samples not correctly identified. True Negatives (TN) are the number of correctly identified negative examples, and False Negatives (FN) are the number of positive samples not correctly identified. The calculation formulas are as follows:


$$Acc = \frac{{TP + TN}}{{TP + FP + FN + TN}}$$

$$P = \frac{{TP}}{{TP + FP}}$$

$$R = \frac{{TP}}{{TP + FN{\ }}}$$

$${F_1} = 2 \times \frac{{P \times R}}{{P + R}}$$

Additionally, the Matthews correlation coefficient (MCC) is a measure of the performance of a classifier, especially in cases of unbalanced datasets. It is a value between −1 and +1, where +1 represents perfect prediction, 0 indicates random prediction, and −1 means completely incorrect prediction. MCC considers all four types of outcome. Therefore, it is considered a very balanced measure of performance, capable of reflecting the performance of a classifier in a single number. Its calculation formula is:


$$MCC = \frac{{TP \times TN - FP \times FN}}{{\sqrt {\left( {TP + FP} \right)\left( {TP + FN} \right)\left( {TN + FP} \right)\left( {TN + FN} \right)} }}$$

###### Baselines

In our study, the primary data characteristic of focus is short text. In the model consideration part, we looked at models that have demonstrated the ability to handle short texts. Specifically, we selected three types of neural network models, FastText, TextCNN, and BiLSTM + Attention, as well as four traditional machine learning models, Logistic Regression, Random Forest, SVM, and Naive Bayes, as our baselines. These models are widely regarded as performing well in handling short text data [21–27]. By comparing ACUBERT with these classic and modern models, we aim to comprehensively assess the efficacy and advantages of ACUBERT in handling short-text data in TCM and acupuncture.

###### Experiments setup

In this process, we adopt a supervised learning approach, utilizing the optimized ACU-IKD dataset, randomly divided into a training set, test set, and validation set at a ratio of 8:1:1. Regarding model implementation, we used the PyTorch platform and Python 3.9 to realize the proposed ACUBERT and related models. Specifically, the configuration of the ACUBERT model is, as shown in Table 3, to facilitate practical training. Moreover, for ACUBERT and all selected baseline models, we set a uniform initial learning rate of 2e-5 and compared their performance after conducting training for 10 epochs. This approach allowed us to effectively gauge the strengths and limitations of ACUBERT in contrast to the baseline models in handling the classification of acupuncture indication entities.

**Table 3.** Model parameter setting table

Parameter	Numerical value
Hidden_size	768
Transformer blocks	12
Number of attention heads	12
Feed-forward	3072
Dropout	0.5
Sentence_length	128
Optimizer	Adam
Batch_size	32

Show the parameter settings employed by the ACUBERT model is trained on optimized ACU-IKD dataset. It details the parameters such as hidden size, number of transformer blocks, attention heads, feed-forward network size, dropout rate, sentence length, optimizer type, and batch size.

## Results

In this section, we will precisely detail the comparative results of ACUBERT against other models. Following this, we discuss the optimization process carried out on the ACUBERT model through algorithm tuning and its impact. Finally, by demonstrating ACUBERT’s classification performance across various specific categories, we validate its performance and advantages in different aspects.

### Model performance comparison

We tested different models using the optimized ACU-IKD dataset. The comparative results in Table 4 show that the ACUBERT model performs well when processing this dataset, especially in balancing P and R. In comparison, traditional machine learning models such as Logistic Regression, Naive Bayes, SVM, and Random Forest performed lower than ACUBERT on all metrics. The likely reason for this disparity is that these models struggle to effectively handle the characteristics of the acupuncture indication entities, such as short-text length and feature sparsity, preventing them from fully capturing the text data’s complexity and deep semantic information. Other neural network (NN) models, such as FastText, TextCNN, and BiLSTM + Attention, outperform traditional machine learning methods in terms of performance. This superiority is due to the powerful learning capability of NN models, which require far fewer features compared to conventional machine learning.

**Table 4.** Comparison results of different models trained on the same dataset

Model	Accuracy	Precision	Recall	F_1_
ACUBERT	0.80	0.82	0.81	0.81
Logistic regression	0.60	0.68	0.58	0.60
Naive Bayes	0.57	0.62	0.55	0.56
SVM	0.63	0.74	0.58	0.62
Random Forest	0.60	0.68	0.56	0.59
FastText	0.71	0.71	0.65	0.67
TextCNN	0.79	0.82	0.77	0.78
BiLSTM + Attention	0.81	0.81	0.78	0.79
ACUBERT*	**0.83**	**0.84**	**0.83**	**0.83**

Note: Bold values represent the best performance among all the methods. ACUBERT* is the model optimized for algorithm tuning parameters.

However, the performance of these models still falls short of ACUBERT. Such a comparison highlights the advantages of ACUBERT in specific tasks and the effectiveness of its adjustment strategies. Overall, ACUBERT demonstrated a significant performance advantage over other models in handling feature sparsity and short text data on the same dataset. This achievement validates the strong potential of deep bidirectional transformer models in understanding complex medical texts and extracting essential information. It opens new research directions and possibilities for acupuncture and traditional medicine in NLP.

### Algorithm hyperparameter optimization

In our research, we performed hyperparameter tuning on the ACUBERT model to better identify and classify acupuncture indication entities. Initially, following the recommendations of the original BERT paper [46], we set the Epochs to 10 and the learning rate (lr) to 2e-5. However, during the experiments, we found that the model converged slowly and exhibited low learning efficiency, tending towards an underfitting state. Underfitting [47] refers to the model’s inability to fit the training data well and capture the accurate patterns in the data, analogous to an acupuncturist lacking practical treatment experience and being unable to diagnose and treat patients accurately. Therefore, we adjusted the lr. After increasing the lr to 4e-5, the model’s R and F1 scores improved, as shown in Table 5. Expanding the lr to 5e-5 did not result in significant further improvements in model performance. Based on Occam’s Razor principle [48], we eventually settled on a lr 4e-5. This approach helped balance learning efficiency and model accuracy, optimizing the model’s performance for our specific application in meridian entities classification of acupuncture indication.

**Table 5.** Status and reasons for setting modifications before and after parameter tuning

Parameter	Learning rate	Epoch
Initialize	2^e−5^	10
	*P*:0.82	*P*:0.82
First training result	*R*:0.81	*R*:0.81
	*F_1_*:0.81	*F_1_*:0.81
Parameter tuning settings	4^e−5^	5
	P:0.82	*P*:0.84
Adjusted result	R:0.82	*R*:0.83
	*F_1_*:0.82	*F_1_*:0.83

Including the initial setting of learning rate and epoch, first training result, parameter tuning settings, and adjusted result, covering *P, R*, and *F_1_*.

**Figure 2.**
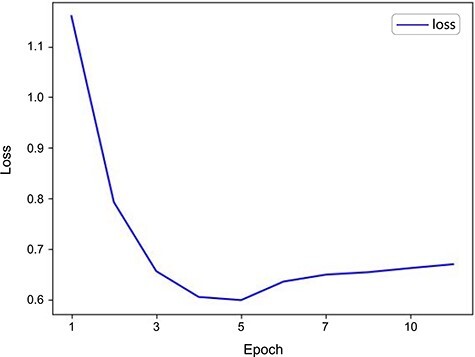
Validation loss curve for the ACUBERT model.

When validating the model’s performance, we paid particular attention to the loss changes on the validation set, as shown in Fig. 2. The results indicated that the model had begun to overfit by the time Epoch reached 10. Overfitting [49] means the model performs excellently on training data but poorly on unknown data, akin to an acupuncturist who relies solely on textbook cases in practice but struggles with new, real-life circumstances. This is because the model is overly sensitive to noise and details in the training data, leading to overfitting. We observed a progressive decline in loss during training, which signifies an enhancement in the model’s capabilities. Notably, the loss reached its nadir at the 5th epoch (loss = 0.65), indicating the model’s peak predictive ability [50]. After that, the loss began to rise and stabilize, indicating the onset of overfitting. Consequently, we set the Epoch to 5 to achieve optimal model performance. This adjustment enhanced the model’s P, R, and F1 scores significantly. As shown in Table 4, the ACUBERT model excels in Acc, P, R, and F1 scores, achieving optimal results in all metrics. Additionally, after hyperparameter optimization, the ACUBERT model achieved a MCC of 0.82, reflecting the model’s comprehensive ability to correctly identify positive classes and avoid wrongly marking antagonistic classes, indicating excellent performance distinguishing between different categories.

### Performance evaluation of the ACUBERT model in meridian category recognition

In testing the classification of meridian categories, we employed the ACUBERT model with optimized parameters. The detailed outcomes are presented in Table 6. The data reveals that ACUBERT demonstrates substantial consistency and reliability in identifying acupuncture indication entities across various meridian categories. With an average P, R, and F1 score of ∼0.82 across the 15 meridian categories, the model’s performance indicates its stability in pinpointing indication in acupuncture and its uniform efficacy across diverse categories. Notably, the model exhibited enhanced P and R in the lung meridian (LU) and large intestinal meridian (LI), achieving 0.88 and 0.95, respectively. These findings suggest that ACUBERT can precisely recognize specific acupuncture indication categories and is proficient in differentiating and identifying associated meridian indications.

**Table 6.** Meridian labeling results based on ACUBERT

Category	Number of acupoints	Proportion to all meridians (%)	Precision	Recall	F1
LU	10	2.79	0.88	0.83	0.85
LI	19	5.29	0.83	0.95	0.89
ST	45	12.53	0.94	0.87	0.90
SP	21	5.85	0.77	0.75	0.76
HT	9	2.51	0.79	0.69	0.73
SI	19	5.29	0.89	0.70	0.78
BL	66	18.38	0.73	0.84	0.78
KI	27	7.52	0.89	0.89	0.89
PC	9	2.51	0.78	0.88	0.82
TE	23	6.41	0.83	0.83	0.83
GB	44	12.26	0.78	0.84	0.81
LR	14	3.90	0.84	0.85	0.85
CV	24	6.69	0.85	0.76	0.81
GV	29	8.08	0.70	0.78	0.74
NM	0	0.00	0.78	0.86	0.82
Mean value	23.93	6.67	0.82	0.82	0.82

Abbreviations: LU, lung meridian of hand-Taiyin; LI, large intestine meridian of hand-Yangming; ST, stomach meridian of foot-Yangming; SP, spleen meridian of foot-Taiyin; HT, heart meridian of hand-Shaoyin; SI, small intestine meridian of hand-Taiyang; BL, Taiyang bladder meridian of foot; KI, kidney meridian of foot-Shaoyin; PC, pericardium meridian of hand-Jueyin; TE, triple energizer meridian of hand-Shaoyang; GB, gallbladder meridian of foot-Shaoyang; LR, liver meridian of foot-Jueyin; CV, conception vessel; GV, governor vessel; NM, not prominent meridians characteristics.

However, the ACUBERT model showed weaker performance in recognizing indications associated with the Foot Taiyang Bladder Meridian (BL) and the Governor Vessel (GV), with P and R slightly below the average. This performance difference could be attributed to several factors: on the one hand, these meridians may have more complex characteristics involving a wider variety of indications, posing more significant challenges for the model’s learning and recognition. On the other hand, a lack of sufficient samples could also be an essential factor. If these meridians have a relatively minor number of samples in the training data, it might lead to inadequate learning of these categories’ features, thereby impacting recognition performance.

The universality and consistency of the model across various meridian categories further prove the practicality and effectiveness of the ACUBERT model in recognizing entities in acupuncture medical texts. Overall, the results indicate that ACUBERT shows significant potential in handling the complex task of acupuncture indication classification, particularly demonstrating significant application value in dealing with distinctly featured meridian categories.

## Discussion

### Analysis of the ACUBERT model performance

In this study, we developed the ACUBERT model, a BERT-based enhancement focused on identifying and classifying acupuncture-related meridian indications. BERT is a pretrained language representation model [51], through a self-supervised learning method, learns efficient feature representations for words from a vast corpus [52], demonstrating exceptional capabilities, particularly in handling short texts with feature sparsity and data mismatch issues—precisely, the needs of our research. The innovation of the ACUBERT model lies in its specific targeting of professional vocabulary in TCM acupuncture for the recognition and classification of short-text entities. Through comparisons with various traditional machine learning models, deep learning models, and pre-trained models, the ACUBERT model has significantly improved accuracy in entity classification tasks, reaching nearly 85%. This achievement enhances the feasibility of classifying professional acupuncture terminology and demonstrates the model’s potential for practical application.

Machine learning is a crucial component of this experiment. Due to the rich prior knowledge available, fine-tuning based on default parameters has proven sufficient to achieve favorable results [53, 54]. This prior knowledge stems from an in-depth analysis of data features and empirical judgment, aiding in making more appropriate choices within traditional machine learning methods. Additionally, in exploring various deep learning approaches such as FastText, TextCNN, and BiLSTM + Attention, the attention mechanism-equipped BiLSTM + Attention model exhibited superior performance. This is attributed to the attention mechanism’s ability to address long-term dependencies between inputs and outputs [55]. In comparing BERT and BiLSTM + Attention, ACUBERT, based on the Transformer architecture and utilizing a self-attention tool, achieves efficient parallel computation, enhancing processing speed [56]. Compared to the recurrent neural network (RNN) underlying structure of BiLSTM + Attention, the Transformer can attend to all input parts simultaneously, improving efficiency and accuracy in information extraction compared to the sequential processing of RNNs [57]. It learns more from limited information, effectively overcoming the shortcomings of short-text lengths. Traditional RNN models tend to overfit when faced with limited training data [58]. The ACUBERT model’s architecture and training methods are tailor-made to extract meaningful patterns from acupuncture datasets, which are often characterized by small sample sizes, incomplete data, and limited diversity. This representation method allows for more precise expression of similar entities, as researchers like Subakti *et al*. [59] have noted, with BERT-based text representation techniques tightening the relationship between similar texts. This is particularly important when dealing with data of complex structures or meanings, as it enhances the model’s ability to recognize and differentiate subtle differences, thereby improving overall accuracy and efficiency [60].

The ACUBERT model has demonstrated exceptional performance in meridian entity classification for acupuncture indications, especially notable in handling feature sparsity and short-text data. This achievement highlights the immense potential of deep bidirectional transformer models in comprehending complex TCM acupuncture texts and extracting critical information. The ACUBERT model’s capability to interpret intricate acupuncture medical texts and capture subtle semantic features stands out, providing valuable insights for intelligent acupuncture diagnostics and treatments.

### Innovative ACU-IKD: characteristics and advantages

Meridians and collaterals are central to TCM and acupuncture, possessing dual value in theory and clinical practice. Within the data-driven framework of modern scientific research, standardized corpora constitute one of the fundamental bases enabling artificial intelligence technologies to delve into acupuncture research and further advance its theoretical understanding [61]. Presently, there is an international lack of research reports in this area.

In this study, we established the world’s first principal treatment diseases database for acupuncture. This dataset exhibits several notable advantages:

Extensiveness: The dataset incorporates a range of essential acupuncture medical books from ancient and modern times, ensuring the source material’s broad coverage and historical depth.

Authoritativeness: All personnel involved in data annotation hold national medical practitioner certificates, guaranteeing the professionalism and accuracy of the annotations (the paper’s appendix provides a complete list of medical qualifications).

Compatibility: The dataset includes clinical case records, teaching materials, and various treatment plans recognized by industry experts, ensuring its practicality and comprehensiveness.

TCM Characteristics: We have increased the number of manually annotated entities to 7000 and provided a rich array of labels and attributes specific to Chinese medicine and acupuncture. We categorize all 54 593 entities into 14 classes (corresponding to the fourteen meridians), and we reserve a classification (the 15th category: indeterminate meridian attributes) for entities with indeterminate properties.

Moreover, in response to the challenges posed by the brevity of text data and the limited feature dimensions, we effectively enhanced the model’s training and prediction accuracy in this study by constructing a validation set similar in distribution to the test set and adopting a fine-grained annotation strategy. This process demonstrated the importance of data preprocessing and model tuning in improving classification performance and aligned with the findings of Vargas *et al*. [62] in their systematic study on imbalanced data preprocessing techniques for machine learning. Their research highlights the critical role of data preprocessing and model tuning in machine learning applications, especially when dealing with imbalanced datasets. Furthermore, our dataset exhibited impressive performance across multiple deep-learning models (Table 4), particularly in classifying acupuncture indication entities and meridian pattern recognition. This achievement not only attests to the high quality of our database but also offers new perspectives and methodologies for future research in short-text processing, especially in TCM and acupuncture. It contributes to better text classification and understanding while enhancing the model’s accuracy in handling specific domain classification tasks.

### Meridian attribute classification with deep learning: modern technological validation of TCM acupuncture patterns

Classification of meridian attributes, or meridian affiliation, is a distinctive aspect of TCM acupuncture and one of the core features that differentiate acupuncture from the traditional TCM practice of “Da Fang Mai” (internal medicine). The characteristics of this meridian classification are not uniformly distributed naturally for various reasons.

Firstly, there’s a significant disparity in the number of acupoints each meridian contains, ranging from the most in the Foot Taiyang Bladder Meridian (BL), which has 66 points, to the least in the Hand Shaoyin Heart Meridian (HT) and Hand Jueyin Pericardium Meridian (PC), which has 9 points each. This results in a significant variation in the frequency of acupoint usage in medical texts, particularly in acupuncture teaching materials.

Secondly, there’s a variation in the number of annotations: the definition of meridian characteristics for principal acupuncture treatments is somewhat ambiguous. Even when referring to the “Huangdi Neijing,” considered a standard, it contains only 236 records, of which not all are sufficiently detailed for comprehensive annotation. From this perspective, the differentiation between meridians itself is not inherently high.

In addition, the number of meridian characteristic values varies significantly, with some corresponding to 9 pathological conditions while others up to 26. These factors collectively impact the efficiency and accuracy of meridian classification. For example, despite the BL accounting for the highest proportion of acupoints (18.38%), it does not have the highest recall or precision rates. This phenomenon suggests that the number of acupoints in a meridian does not always correlate with its clinical efficacy. It also presents a nonlinear relationship between the number of acupoints and the indications they treat. The therapeutic effect of acupoints is not merely improved with increasing quantity. Still, it is closely related to their position, function in the meridian system, and the synergistic effect with other acupoints, requiring in-depth data analysis to elucidate these complex relationships. For example, the high P and F1 scores of the Foot Yangming Stomach Meridian (ST) underline its close connection with various diseases, supporting the TCM theory of “the stomach being the foundation of the human body” and implying its significant role in treating multiple conditions. The frequent appearance and efficiency of ST acupoints, such as Zusanli (ST36), in analyzing acupoint compatibility rules of acupuncture clinical prescriptions may also indirectly prove this theory [63, 64].

Overall, ACUBERT has achieved average P, R, and F1 scores of over 0.8 across all meridians, demonstrating a fundamental capability for indication differentiation and meridian attribution. Our ACUBERT model, developed through machine learning methods, has verified the specificity of meridian attribution in a comprehensive database of acupuncture indication, marking the first attempt across languages, eras, and schools, showcasing its potential in the acupuncture field for acupuncture indication identification and meridian attribution classification. These findings not only deepen our understanding of the complexity of the meridian system but also provide new perspectives and tools for the modernization and scientization of acupuncture.

### Prospects for research

Meridian pattern differentiation is a crucial aspect of the multidimensional approach to acupuncture diagnostics. While preliminary achievements have been made in machine learning, several key challenges remain. Specifically, the issues in the intelligent research of acupuncture diagnostics primarily stem from three areas:

The complexity of the research subject: the field of acupuncture diagnostics encompasses various content, including multiple practitioners, schools of thought, and acupuncture texts from different periods. These texts record a diverse range of information, such as compilations of experience and clinical case studies. However, this diversity challenges the intelligent analysis of acupuncture texts, particularly in knowledge extraction. Research often limits itself to specific diseases or individual practitioners, with different teams frequently addressing similar issues. This not only highlights the inconsistency in knowledge representation due to the complexity of the subject matter but also becomes a significant factor hindering further research development. Intelligent analysis of acupuncture texts relies on a unified framework to advance the study of acupuncture diagnostics. Developing and adopting a standardized dataset for acupuncture texts is crucial to address this issue. It can effectively streamline the subject matter and enhance research efficiency.

Lack of a Structured Analysis System: Current research primarily focuses on symptom-pattern differentiation algorithms and acupoint selection based on symptoms, revolving mainly around the traditional internal medicine system of Chinese Medicine. Researchers also center the corresponding algorithms and model training on these aspects [65–67]. However, acupuncture texts inherently contain complex and sparse information. Methodological limitations of the analysis system mean that even though some studies have begun addressing “hidden nodes” and “implicit knowledge,” the lack of a structured analysis system suitable for acupuncture diagnostics often reduces the results to retrospective summaries of existing clinical data or case records. This issue makes it challenging to develop a universal intelligent acupuncture diagnostic application. To overcome this limitation, there is an urgent need to establish a structured analysis framework tailored to acupuncture diagnostics to enhance the depth and universality of research applications.

Acupuncture Diagnostics Knowledge Requires Updating and Reconstruction: While some studies have applied advanced information technology methods, they have overlooked the critical aspects of acupuncture diagnostics. These studies directly research the correlations between acupoints and symptoms, an incomplete approach. Achieving a more comprehensive integrated diagnostic analysis necessitates combining traditional acupuncture knowledge with modern medical symptom information. We need to update and reconstruct our existing understanding of acupuncture diagnostics to adapt it to the contemporary technological context better and improve its responsiveness to clinical needs. Optimizing the knowledge’s hierarchy and logic will further promote the high-quality development of the field of acupuncture.

## Conclusion

This study utilizes the well-established and widely recognized BERT model from NLP to conduct machine learning research on the meridian attributes of acupuncture indications. The proposed ACUBERT model surpasses baseline models in classifying meridian syndrome entities. It incorporates TCM principles, specifically the eight principles of differentiation and zang-fu differentiation, to train a meridian differentiation model and establish an acupuncture indication corpus with TCM characteristics. As the inaugural deep learning exploration into acupuncture meridian differentiation, this study sets a new benchmark, offering reproducible results and robust methodologies for future research. This work forms a crucial foundation for the development of intelligent diagnostics in acupuncture.

## Supplementary Material

baae083_Supp

## Data Availability

All required material is contained in the Supplementary material.
