# Supplementary material for: Acupuncture indication knowledge bases: meridian entity recognition and classification based on ACUBERT
Source: Database (Oxford). 2024 Aug 30;2024:baae083. doi: 10.1093/database/baae083 (PMC11363959; doi:10.1093/database/baae083)
Supplement: baae083_Supp [file baae083_supp.zip › suppl_data/patent.pdf]

国家知识产权局

210019

江苏省南京市建邺区奥体大街 68 号 5A 栋 9 层 江苏瑞途律师事务所
金龙(025−85803161)

发文日：

2023 年 10 月 05 日

申请号或专利号：202310451949.2 **发文序号：**2023100500163950

申请人或专利权人：南京中医药大学 南京柯基数据科技有限公司

发明创造名称：一种针灸知识图谱构建方法、装置及计算机设备

发明专利申请公布通知书

上述专利申请，经初步审查，符合专利法实施细则第 44 条的规定。根据专利法第 34 条的规定，该申请在 39 卷 3902 期 2023 年 09 月 29 日专利公报上予以公布。

提示：

1.发明专利申请人可以自申请日起 3 年内提交实质审查请求书、缴纳实质审查费，申请人期满未提交实质审查请求书或期满未足额缴纳实质审查费的，该申请被视为撤回。

2.专利费用可以通过网上缴费、银行/邮局汇款、直接向代办处或国家知识产权局专利局缴纳。缴费时应当写明正确的申请号/专利号、费用名称及分项金额，未提供上述信息的视为未办理缴费手续。了解缴费更多详细信息及办理缴费业务，请登录国家知识产权局官方网站。

3.申请人可以访问国家知识产权局政府网站（www.cnipa.gov.cn），在专利检索栏目中查询公布文本。如果申请人需要纸件申请公布单行本的纸件，可向国家知识产权局请求获取。

4.申请文件修改格式要求：

对权利要求修改的应当提交相应的权利要求替换项，涉及权利要求引用关系时，则需要将相应权项一起替换补正。如果申请人需要删除部分权项，申请人应该提交整理后连续编号的部分权利要求书。

对说明书修改的应当提交相应的说明书替换段，不得增加和删除段号，仅只能对有修改部分段进行整段替换。如果要增加内容，则只能增加在某一段中；如果需要删除一个整段内容，应该保留该段号，并在此段号后注明：“此段删除”字样。段号以国家知识产权局回传的或公布/授权公告的说明书段号为准。

对说明书附图修改的应当以图为单位提交相应的替换附图。

对说明书摘要文字部分修改的应当提交相应的替换页。对摘要附图修改的应当重新指定。

同时，申请人应当在补正书或意见陈述书中标明修改涉及的权项、段号、图、页。

审 查 员：自动审查 审查部门：初审及流程管理部

联系电话：010−62356655

国家知识产权局 专利审查业务章 1101081359734

纸件申请，回函请寄：100088 北京市海淀区蓟门桥西土城路 6 号 国家知识产权局专利局受理处收
电子申请，应当通过专利业务办理系统以电子文件形式提交相关文件。除另有规定外，以纸件等其他形式提交的文件视为未提交。
