# Supplementary material for: Acupuncture indication knowledge bases: meridian entity recognition and classification based on ACUBERT
Source: Database (Oxford). 2024 Aug 30;2024:baae083. doi: 10.1093/database/baae083 (PMC11363959; doi:10.1093/database/baae083)
Supplement: baae083_Supp [file baae083_supp.zip › suppl_data/Supplementary materials-Table S1.docx]

**Table S1 List of acupuncture and moxibustion medical books**

| **Number** | **Names of the acupuncture books in original language** | **Author(s)** | **Publication Years** | **ISBN** | **Language** |
| --- | --- | --- | --- | --- | --- |
| **1** | 大明琢周针法一轴（匹地流） | 匹地喜庵 | 1679 | 9787513248464 | Chinese Translation of Japanese |
| **2** | 针灸溯洄集 | 高津松悦斋 | 1694 | 9787513248464 | Chinese |
| **3** | 杉山真传流（奥虎龙之卷） | 岛浦和田一 | 1709 | 9787513248464 | Chinese Translation of Japanese |
| **4** | 杉山真传流（表之卷） | 岛浦和田一 | 1709 | 9787513248464 | Chinese Translation of Japanese |
| **5** | 杉山真传流（中卷） | 岛浦和田一 | 1709 | 9787513248464 | Chinese Translation of Japanese |
| **6** | 灸炳要览 | 堀元厚 | 1723 | 9787513248464 | Chinese Translation of Japanese |
| **7** | 灸点图解 | 香川舆司马 | 1756 | 9787513248464 | Chinese Translation of Japanese |
| **8** | 针灸则 | 菅沼周圭 | 1766 | 9787513248464 | Chinese Translation of Japanese |
| **9** | 熙载录 | 垣本针源 | 1782 | 9787513248464 | Chinese Translation of Japanese |
| **10** | 名家灸选三编 | 和气惟亨，平井庸信 | 1805 | 9787513248464 | Chinese Translation of Japanese |
| **11** | 针灸说约 | 石坂宗哲 | 1811 | 9787513248464 | Chinese Translation of Japanese |
| **12** | 困学穴法 | 石塚汶上 | 1835 | 9787513248464 | Chinese Translation of Japanese |
| **13** | 针论 | 葛西清 | 1862 | 9787513248464 | Chinese |
| **14** | 百法针灸 | 杉山和一 | 1932 | 9787513248464 | Chinese Translation of Japanese |
| **15** | 针灸秘开 | 玉森贞助 | 1958 | 9787513248464 | Chinese Translation of Japanese |
| **16** | 内科针灸治疗学 | 邱茂良 | 1970 | 9787534567865 | Chinese |
| **17** | 黄帝明堂经辑校 | 黄龙详 | 1987 | 9787506700214 | Chinese |
| **18** | 针灸逢源 | 李学川 | 1987 | 7532300196 | Chinese |
| **19** | 中国针灸治疗学 | 邱茂良 | 1988 | 7534504015 | Chinese |
| **20** | 常见病症的针灸辨证施治 | 徐恒泽 | 1988 | 7117008628 | Chinese |
| **21** | 中医学问答题库（针灸分册） | 程莘农 | 1988 | 9787800131912 | Chinese |
| **22** | 金匮要略 | 李克光 | 1989 | 9787117007320 | Chinese |
| **23** | 实用针灸选穴手册 | 杨兆民，鞠传军 | 1990 | 9787800222177 | Chinese |
| **24** | 常见病针灸疗法 | 阮少南 | 1990 | 9787533503222 | Chinese |
| **25** | 针治疑难奇症案汇 | 黄宗勖，余昌德 | 1991 | 9787533504533 | Chinese |
| **26** | 普济方 | 朱橚 | 1991 | 9787532509140 | Chinese |
| **27** | 实用针灸治病法精华 | 肖少卿 | 1992 | 7537705887 | Chinese |
| **28** | 中国针灸独穴疗法 | 陈德成 | 1992 | 9787538422580 | Chinese |
| **29** | 乡村常见病的针灸与推拿治疗 | 夏治平，李玉堂 | 1993 | 781010196 | Chinese |
| **30** | 医心方 | 丹波康赖 | 1993 | 9787508000428 | Chinese Translation of Japanese |
| **31** | 中国针灸配穴疗法 | 王庆文、陈德成 | 1995 | 7805844305 | Chinese |
| **32** | 针灸治法与处方 | 邱茂良 | 1995 | 9787532336456 | Chinese |
| **33** | 千金方 | 孙思邈 | 1996 | 9787117022934 | Chinese |
| **34** | 中国针灸处方学 | 肖少卿 | 1998 | 7227016803 | Chinese |
| **35** | 针灸学临床 | 王玲玲 | 2000 | 7810104926 | Chinese |
| **36** | 杨甲三临证论治 | 杨甲三 | 2000 | 9787538835496 | Chinese |
| **37** | 实用针灸学 | 吴旭，盛灿若 | 2001 | 7801571010 | Chinese |
| **38** | 杨甲三中国百年百名中医临床家丛书 | 胡慧 | 2001 | 9787801561473 | Chinese |
| **39** | 黄帝内经 | 王冰 注 | 2003 | 9787801742049 | Chinese |
| **40** | 中国百年百名中医临床家丛书 黄宗勖 | 黄宗勖 | 2004 | 9787801566447 | Chinese |
| **41** | 承淡安针灸经验集 | 项平，夏有兵 | 2004 | 9787532376865 | Chinese |
| **42** | 中华针灸学 | 王玲玲 | 2004 | 9787534539435 | Chinese |
| **43** | 针灸甲乙经 | 皇甫谧 | 2006 | 9787117076913 | Chinese |
| **44** | 针灸聚英 | 高武 | 2006 | 9787117076319 | Chinese |
| **45** | 针灸大成 | 杨继洲 | 2006 | 9787117076340 | Chinese |
| **46** | 中国针灸治疗学 | 承淡安 | 2006 | 9787533527464 | Chinese |
| **47** | 针灸学.高等医学院校选用教材 | 冀来喜 | 2006 | 9787030089465 | Chinese |
| **48** | A Manual of ACUPUNCTURE | Peter Deadman, Mazi n Al-Khafaji | 2007 | 9780951054659 | English |
| **49** | 新针灸学 | 朱涟 | 2008 | 9787807630999 | Chinese |
| **50** | 黄鼎坚针灸临证经验集要 | 赵利华 | 2008 | 9787117106313 | Chinese |
| **51** | 针灸中药临床学 | 杨长森 | 2008 | 9787117103053 | Chinese |
| **52** | 杨兆民针灸临床经验集萃 | 杨兆民 | 2008 | 9787117103534 | Chinese |
| **53** | 肥胖病的针灸治疗 | 刘志诚 | 2008 | 9787117102216 | Chinese |
| **54** | 针灸真髓 | 代田文志 | 2008 | 9787507730883 | Chinese Translation of Japanese |
| **55** | Yamamoto new scalp acupuncture | Toshikatsu Yamamoto, Helene Yamamoto | 2008 | 4895891690 | English |
| **56** | 实用针灸独穴疗法 | 甘君学 | 2009 | 9787534568350 | Chinese |
| **57** | 实用针灸学 | 石学敏 | 2009 | 9787802317161 | Chinese |
| **58** | 谢锡亮灸法医案 | 谢锡亮 | 2010 | 9787509135525 | Chinese |
| **59** | Chinese Acupuncture & Moxibustion | 程莘农 | 2010 | 9787119059945 | English |
| **60** | 肘后备急方 | 葛洪 | 2011 | 9787530828663 | Chinese |
| **61** | 盛灿若六十年针灸临证传薪 | 顾一煌，孙建华 | 2011 | 9787513206655 | Chinese |
| **62** | 诸病源候论 | 巢元方 | 2011 | 9787506748926 | Chinese |
| **63** | 针灸临床治疗学 | 代田文志 | 2011 | 9787507738315 | Chinese Translation of Japanese |
| **64** | 新编实用针灸学（上册） | 孔昭遐，屠佑生 | 2012 | 9787533753993 | Chinese |
| **65** | 新编实用针灸学（下册） | 孔昭遐，屠佑生 | 2012 | 9787533753993 | Chinese |
| **66** | 杨长森针灸学讲稿 | 杨长森 | 2012 | 9787117164115 | Chinese |
| **67** | 针灸治疗学（十二五教材） | 杜元灏，董勤 | 2012 | 9787117160452 | Chinese |
| **68** | 盛灿若针灸临证精华集 | 陈理，王和生 | 2013 | 9787117180290 | Chinese |
| **69** | 吴旭杂病针灸治验录 | 孙建华，陆斌 | 2014 | 9787117197724 | Chinese |
| **70** | 邱茂良中国百年百名中医临床家丛书 | 何崇 | 2014 | 9787513217880 | Chinese |
| **71** | 承淡安伤寒论新注 | 承淡安 | 2015 | 9787547827024 | Chinese |
| **72** | 国医大师程莘农临证指针 | 王红伟，冯春祥，王贵春 | 2016 | 9787507747157 | Chinese |
| **73** | 筋针疗法 | 刘农虞，刘恒志 | 2016 | 9787117222921 | Chinese |
| **74** | Medical Acupuncture: A Western Scientific Approach | Jacqueline Filshie | 2016 | 9780702043079 | English |
| **75** | 针灸寻真:曾天治针灸学术经验集 | 曾天治 | 2017 | 9787535966964 | Chinese |
| **76** | Neuropuncture-A CLINICAL HANDBOOK OF NEUROSCIENCE ACUPUNCTURE | Michael D. Corradino | 2017 | 9781848193314 | English |
| **77** | 经络腧穴学 | 胡玲、刘清国 | 2018 | 9787547840030 | Chinese |
| **78** | 一针一乾坤 跟师国医大师程莘农笔记 | 常佩芬，常阿喜 | 2018 | 9787500278405 | Chinese |
| **79** | 程莘农 中国针灸学第五版 | 程莘农 | 2019 | 9787117271707 | Chinese |
| **80** | 留章杰研究 | 孟宪军 | 2020 | 9787571304836 | Chinese |
| **81** | 针灸治疗学 | 杨长森 | 2021 | 9787532304943 | Chinese |
| **82** | 针灸资生经 | 王执中 | 2021 | 9787521422269 | Chinese |
